# Taking the lead from our colleagues in medical education: the use of images of the in-vivo setting in teaching concepts of pharmaceutical science

**DOI:** 10.1186/s40545-017-0110-1

**Published:** 2017-07-17

**Authors:** Louise E. Curley, Julia Kennedy, Jordan Hinton, Ali Mirjalili, Darren Svirskis

**Affiliations:** 1 School of Pharmacy, Faculty of Medical and Health Sciences, University of Auckland, Private Bag 92019, Auckland, 1142 New Zealand; 2 Department of Anatomy with Radiology, Faculty of Medical and Health Sciences, University of Auckland, Auckland, New Zealand

**Keywords:** Pharmaceutical sciences teaching, Oral dosage forms, In-vivo imaging, In-vivo location, Disintegration

## Abstract

Despite pharmaceutical sciences being a core component of pharmacy curricula, few published studies have focussed on innovative methodologies to teach the content. This commentary identifies imaging techniques which can visualise oral dosage forms in-vivo and observe formulation disintegration in order to achieve a better understanding of in-vivo performance. Images formed through these techniques can provide students with a deeper appreciation of the fate of oral formulations in the body compared to standard disintegration and dissolution testing, which is conducted in-vitro. Such images which represent the in-vivo setting can be used in teaching to give context to both theory and experimental work, thereby increasing student understanding and enabling teaching of pharmaceutical sciences supporting students to correlate in-vitro and in-vivo processes.

## Background

There is a growing body of literature that demonstrates that the use of visual methods for teaching helps to enhance students learning, including retention of complex information in subjects such as pharmaceutical science and pharmacology [1, 2]. In previous literature authors have presented some innovative methods for teaching pharmaceutical science in pharmacy education. These methods have included using interactive digital images of products [2] and the use of animated videos to illustrate oral solid dosage form manufacturing [3]; both examples have demonstrated improved student learning and favourable perceptions. Other studies have used video modules and clips in a drug assay course, leading to similar improvements in outcomes and perceptions of students [1].

Pharmaceutical sciences is a core element of pharmacy education, and is regarded by the Accreditation Council for Pharmacy Education (ACPE) as an essential element to the development of pharmacists [4]. In addition, The Center for Advancement of the Pharmacy Education (CAPE) educational outcomes stress the importance in foundational pharmaceutical sciences to “solve therapeutic problems and advance patient centred care” [5].

The challenges remains in pharmaceutical science education to find effective teaching strategies. The topics and concepts in pharmaceutical science can be dry and application of these concepts can be difficult for students to conceptualize as teaching is often supported by in-vitro teaching laboratories. As educators we have a duty to ensure that this teaching inevitably has a patient-care focus.

We recognise that there is paucity of research that surrounds enhancing learning in pharmaceutical science education, and therefore this commentary reflects on what we can learn from our colleagues in other healthcare professional programmes to 1) enhance teaching of pharmaceutical sciences by other visual methods, and 2) make our teaching of pharmaceutical sciences more patient-focussed.

Worldwide many medical curricula have used advances in diagnostic imaging to enable students to visualise the structure and function of human anatomy [6, 7]; for example, all medical schools in Australia and New Zealand [8] and 92% of medical schools in Canada [9] use imaging to some extent to teach anatomy. A recent review conducted by Grignon and colleagues (2016) evaluated the role of imaging in teaching anatomy [10]. Whilst a minority of papers in the review showed no differences in outcomes for students, the majority of cases found that imaging enhanced the quality and efficiency of instruction in human anatomy [10].

Medical images have been used alone and as a synergistic teaching tool alongside cadaveric dissection for teaching anatomy [6], with studies reporting positive effects on learning; specifically improvements in identification of anatomical structures [11] and long term knowledge retention [12]. Using imaging in anatomy teaching allows for a unique understanding and visualisation of the spatial relationship between structures and for the future ability to recognise anatomical structures on medical images. For example the teaching of anatomy using ultrasound (US) was reported by Brown et al. [13] and Dreher et al. [14] to result in an increase in the confidence of students in anatomical recognition, which subsequently correlated with performance on tests of skills and knowledge. In addition, combining US images with cross-sectional anatomy teaching improved students confidence more than US images alone [15].

The principle of using imaging in teaching methodology does not have to be limited to medical curricula; similar techniques could be used in learning environments to visualise and compare the behaviour of pharmaceutical formulations alongside standard benchtop experiments. However to date, a search of the literature yields no published study where this type of imaging has been used to teach pharmaceutical science. We do recognise however, that such images may be being used in teaching, with no research surrounding their use.

Imaging techniques have the potential to increase our understanding of in-vivo conditions, and the subsequent impact on dosage formulations, which also may give context to the current experimental setups being used to mimic in-vivo conditions. The use of imaging techniques to further our knowledge of the in-vivo behaviour of drug formulations is of increasing interest in research and development [16, 17]; images resulting from these techniques could also be used to enhance teaching practice.

An example would be learning surrounding oral administration of formulations. Oral administration is notably the most preferred and frequently used route for drug therapy [18] and is often focussed on during pharmaceutical science education due to overall patient acceptability and being the most frequently used route. The physiological factors and formulation design have significant effects on the disintegration, dissolution and absorption of the drug [19], making it important to understand the in-vivo behaviour of pharmaceutical formulations. In pharmaceutical science education, knowledge surrounding formulating different dosage forms is a key objective. Current methods for the in-vitro simulation of in-vivo environments are not a completely accurate reflection of conditions the dosage form will be exposed to in the body [20], subsequently, in-vivo/in-vitro correlation of disintegration and dissolution is often poor [21, 22].

Whilst our colleagues in undergraduate medical teaching curricula mainly use imaging to identify structure and function of the anatomy, there is a unique opportunity for pharmaceutical science educators to observe the interaction of a medicine within the in-vivo environment. Not all imaging techniques are appropriate to view this interaction and therefore there must be specific considerations to which techniques would be most useful. There are a number of imaging modalities that are currently available including gamma scintigraphy, magnetic resonance imaging (MRI), computed tomography (CT) and x-rays, US and the newer emerging technique of magnetic moment imaging (MMI), for advantages, disadvantages and examples of publications see Table 1. For the purposes of in-vivo visualisation of oral dosage form location and disintegration, gamma scintigraphy MRI, CT and MMI techniques can be used to obtain images to provide innovative and valuable teaching tools for pharmaceutical science education. The most promising tools, in our opinion, which could be used in generating images for teaching to aid understanding of interactions between dosage forms and the in-vivo environment are MRI and magnetic moment imaging.

**Table 1:** Imaging modalities available to visualise oral dosage forms in the body

Imaging technique	Advantages	Disadvantages	Examples of publications where combined with dosage administration
Gamma scintigraphy	• It is possible to determine both disintegration of the formulation and drug release using this method [27]. • Useful to identify the location of the dosage form within a body mass [27].	• Limited topographic information obtained and the use of radioactive materials [28, 29]. • Radiation involved reduces the number of scans a subject can safely undergo, and restricts its use in women and children [25].	• Wilding et al. [27] reviewed the role of gamma-scintigraphy in the visualisation of oral drug delivery. • Teran and colleagues [30] described the dissolution and disintegration profiles of different drug models depending on the radiotracer chosen.
Magnetic Resonance Imaging	• This type of imaging allows high resolution structural images of the desired area to be obtained [19, 31] with high scan volumes [32]. • No radiation exposure [31]. The avoidance of ionising radiation is an important advantage allowing for the possibility of rapid repeated measurements to observe changes following in-vivo administration of dosage forms [33] • Contrast agents can be incorporated in to the oral dosage form to improve the quality of images. Contrast agents can be either positive or negative in their enhancement and allow for oral dosage forms to be distinguished from food or gas bubbles [32].	• The gastrointestinal tract is highly mobile, and tidal breathing results in movement of the abdominal contents, which can lead to the generation of artefacts on MRI images. Although there are simple solutions to get around this issue [34, 20]. • It can be difficult in a fed state to differentiate the formulation from food and gas bubbles [20]. In addition, participants in the fasted state will have very little water in the stomach and small intestine which may lead to poor imaging of the dosage form possibly altering the dissolution rate [20].	• Reviewed by Richardson [24] for the potential for its use to visualise dosage behaviour in-vivo . • MRI used to visualise the formation of the carbonated alginate raft that is formed with the anti-reflux product Gaviscon® [17]. • Liquid emulsions have also been investigated in the stomach using MRI [16]. • Feasibility study that has investigated, with success, the ability to use MRI to compare different dosage formulations of a drug [23].
Computed Tomography and X-rays	• Imaging using radio-opaque substances is able to identify the position of formulations following oral administration.	• Radio-opaque substances are typically needed for imaging with barium sulphate often used once incorporated into the dosage form [35–37], however limited detail of the surrounding soft tissue anatomy is provided. • High contrast resolution images come with a drawback of requiring greater doses of radiation [38]. This reduces the number of scans that can be performed on a single subject.	• Dissolution of tablets was described by Vemula et al. [36] however the detail was limited, and the time course of data acquisition was over several hours at set time intervals, until the tablet was no longer seen. • Lai and colleagues [28] performed a study where they used a combination of single-photon emission computed tomography/computed tomography (SPECT/CT) to visualise the movement of a colon targeting dosage forms from administration to site of release [28]. SPECT/CT was able to show disintegration of the tablet and the position in the GIT at which this was occurring [28].
Ultrasound	• US uses relatively affordable and portable US machines, increasing accessibility, whilst providing real time acquisition [39].	• Limited applicability in pharmaceutical dosage form evaluation, as detection of dosage forms following oral administration can be difficult due to the presence of food or lack of water [40] which can make identification of the dosage form difficult. • The presence of gas in the GIT acts as an effective reflector of US waves, making quality images difficult to obtain and the visualisation of behaviour of the dosage form difficult [32].	• US has been explored as an avenue for in-vivo imaging of oral dosage forms to visualise ingested medications in acute poisoning cases [40]. However, the results from this study highlight the unreliable images generated through US imaging of oral dosage forms in-vivo [40] • US does have utility in some areas of pharmaceutical imaging due to its ability to distinguish between the small intestine and the colon. Kobayashi [41] presented a case study where US was successfully used to detect the location of the patency capsule in a patient who was evaluated to be at risk of capsule retention in capsule endoscopy
Magnetic Moment Imaging	• MMI provides high resolution images both in temporal and spatial dimensions [26], allowing for tracking of dosage forms through the GIT. • This method also has the advantage of its ability to image real time movements of the dosage forms [25].	• Large costs associated with running and maintaining the equipment, and the special requirement that they be housed in a magnetically shielded environment [19, 25].	• Goodman and colleagues showed how the fed state significantly effecting onset and time course of disintegration and the ability to show real time movement of the dosage form [25]

Recently we have published a feasibility study that has investigated, with success, the ability to use MRI to compare different dosage formulations of a drug [23]. Specifically the study compared the images obtained of immediate release, standard release and slow release oral tablets both by benchtop observations of disintegration and using MRI, in both in-vitro and in-vivo settings [23]. Our findings highlighted the differences in in-vitro disintegration, with that of disintegration within the human stomach [23]. In addition to our own research, other studies have successfully used MRI to visualise the formation of the carbonated alginate raft that is formed and floats on top of the stomach contents after ingestion of the anti-reflux product Gaviscon®. The authors of this study demonstrated the use of MRI to assess dynamic changes of rafts in-vivo after being able to identify the duration and positioning of the raft [17]. In addition, the in-vivo behaviour of liquid emulsions have also been investigated in the stomach using MRI [16]. Furthermore, a review by Richardson and colleagues evaluated the use of MRI in both pre-clinical and clinical models, demonstrating that there was a potential for its use to visualise dosage behaviour in-vivo [24].

MMI is a relatively new technique that uses magnetically labelled solid dosage forms which produce a magnetic field from magnetic dipole moments [25]. MMI provides high resolution images both in temporal and spatial dimensions [26], allowing for tracking of dosage forms through the GIT. Weitschies and colleagues [26] used MMI to highlight highly variable transit times in healthy volunteers. This methodology therefore has the potential of visualisation of changes to GIT behaviour, for example, disease states or the effect of fed versus fasted states which may alter drug transition time.

MMI also has the advantage of its ability to image real time movements of the dosage forms [25]. This can allow for visualisation of the passage of the dosage form through the GIT, showing the effects of gastric contents on this movement [25]. This is important as the position of the dosage form in the GIT can affect its disintegration, dissolution [25] and absorption. This has been described by Goodman and colleagues in their study which showed a fed state significantly effecting onset and time course of disintegration [25]. However, despite the clear advantages, there are large costs associated with running and maintaining the equipment required for MMI [19, 25].

The use of such images in pharmaceutical science teaching have the potential to parallel the improvements that have been seen other undergraduate healthcare curricula. These images could be used in a variety of teaching sessions, including lectures and small group teaching sessions. We believe they hold great value to be used in practical sessions alongside in-vitro experiments. For example, running pharmacopoeial disintegration tests with in-vivo images available for comparison at certain time points would highlight the similarities and differences between benchtop experiments and formulation behaviour in a patient’s body following administration. At The University of Auckland’s School of Pharmacy we have undergone a curriculum redevelopment and one objective is to achieve a fully integrated curriculum. One of the strategies is to place more of a focus on patient-centred care in our pharmaceutical science teaching by embedding a series of images acquired by MRI and benchtop visualisation [23] into teaching sessions regarding disintegration and dissolution of different formulation types. Images are used for students to visualise how certain processes occur in the human body, for example MRI images demonstrate disintegration occurring in the human stomach. We believe that images of the in-vivo setting would be beneficial as instructional aids for teaching concepts in pharmaceutical science. The information often being taught combines a drug containing formulation with anatomy, chemistry and clinical pharmacy and these images will act as a way of aiding with the application of knowledge to the patient.

In-vivo images demonstrating the fate of oral dosage forms in the body have been published in various studies detailed in this commentary and can be used in teaching to give context to both theory and experimental work. Such images could be used to increase student understanding and enable teaching of pharmaceutical sciences supporting students to correlate in-vitro and in-vivo processes. Further research is warranted to evaluate the use of these types of visual teaching methods, and whether there are improvements in learning, including application of key principles, and whether students perceive benefits in their usage.
